# Dietary Intake of Masters Athletes: A Systematic Review

**DOI:** 10.3390/nu15234973

**Published:** 2023-11-30

**Authors:** Sheran Guo, Gabriella L. L. Shaoni, Wendy A. Stuart-Smith, Alyse J. Davies, Janelle A. Gifford

**Affiliations:** 1 Discipline of Nutrition and Dietetics, School of Nursing and Midwifery, Faculty of Medicine and Health, The University of Sydney, Camperdown, NSW 2006, Australia; sheran.guo@sydney.edu.au (S.G.); wendy.stuart-smith@acu.edu.au (W.A.S.-S.);; 2 Charles Perkins Centre, The University of Sydney, Camperdown, NSW 2006, Australia; 3 School of Behavioural and Health Sciences, Australian Catholic University, North Sydney, NSW 2060, Australia; 4 Discipline of Exercise and Sport Science, Sydney School of Health Sciences, Faculty of Medicine and Health, The University of Sydney, Camperdown, NSW 2006, Australia; 5 Sport and Physical Activity Research and Teaching Network (SPARTAN), The University of Sydney, Camperdown, NSW 2006, Australia

**Keywords:** masters athletes, sport, dietary intake, macronutrients, micronutrients, healthy ageing

## Abstract

Dietary practices of masters athletes (MAs) may promote healthy ageing; however, they are poorly understood. The aims of this systematic review were to synthesise the literature on the dietary intakes of MAs and undertake comparisons between younger (35–50 years) and older (>50 years) MAs and the general population. A search was conducted across seven databases to identify relevant publications for screening and data extraction. Averages for energy intake (EI), macronutrients, and micronutrients were compared with data from the 2011–2012 Australian Health Survey (general population). Twenty-six studies (*n* = 2819) were included. Energy intake was higher for older (8908 kJ/d versus 7792 kJ/d) but not younger MAs (9073 kJ/d versus 8872 kJ/d) versus the general population. Younger versus older male MAs had higher energy and macronutrient intakes. Energy intake for older was comparable to younger female MAs (7819 kJ/d versus 7485 kJ/d), but older had higher protein, lower carbohydrate, and higher micronutrient intakes. Micronutrient intake was higher in MAs than the general population. Similar EIs for older MAs and younger general population may indicate potential for a higher-quality diet. Younger female MAs may restrict or misreport EI, requiring further investigation. There is a need for more comprehensive assessments of dietary intake in MAs to ascertain diet quality in relation to health.

## 1. Introduction

A variety of physiological changes occur with ageing, including the loss of lean tissue [1] and subsequent declines in resting metabolic rate and physical capacity [2,3]. Without an increase in physical activity, this may result in an altered energy balance due to reduced energy expenditure [4] and energy intake [3] to remain weight-stable. As a result of a lower energy ‘budget’ [5], older individuals are at greater risk of nutritional inadequacy, often exacerbated by poor dietary practices. In the 2011–2012 Australian Health Survey (AHS), Australians aged 51 years and over consumed at least 30% of their energy intake from discretionary sources [6]. Suboptimal dietary intakes, including the consumption of energy-dense, nutrient-poor foods, are a leading risk factor for noncommunicable diseases [7]. This is reflected in the rising prevalence of nutrition-related chronic diseases with increasing age, particularly after 55 years, including cardiovascular diseases, sarcopenia, osteoporosis, and diabetes [8].

Masters athletes (MAs) are a unique group of older adults whose lifestyle behaviours may reduce the risk or burden of chronic disease and/or optimise athletic performance. When compared to the general Australian population, MAs have a lower prevalence of hypertension, type 2 diabetes mellitus, hyperlipidaemia, cancers, and osteoporosis [9]. MAs are a heterogeneous group of physically active individuals, typically aged 35 years or over, or as defined by sports-specific age cut-offs. They exceed population guidelines in their levels of physical activity; additionally, some may engage in systematic training, and some may compete [5,9,10].

To date, much of the existing literature focuses on dietary intakes of younger elite athletes. Little is known about the dietary practices of MAs. Limited evidence suggests that they may consume more nutritionally complete, higher-energy diets to a greater extent than their less active peers [11,12,13,14,15]. Expending more energy through physical activity allows MAs to consume greater quantities of food to meet nutritional requirements, particularly those that increase with age, such as protein and calcium [16,17]. Investigating the dietary practices of MAs can help to inform on strategies to support their health and performance. These strategies may potentially be translated more broadly to the general population.

While dietary studies on MAs do exist, they are often limited to pre-, within-competition, or recovery nutrition strategies, which are unlikely to represent an athlete’s usual diet [12,18,19,20,21]. The extent of dietary analysis may also be limited in the number of nutrients or food groups examined [6,17,22]. Additionally, studies in MAs generally focus on the effects of physical activity and have not been designed to determine or assess nutritional adequacy. This paper has two distinct aims: (1) to synthesise the available evidence on dietary intakes of MAs and (2) to undertake comparative assessments between MAs, the general population, and dietary recommendations. The findings will provide a greater insight into the adequacy of their dietary practices, highlighting their risk profiles compared to the general population. Where applicable, MA dietary strategies may then be utilised in health promotion strategies to encourage healthy ageing in the general population.

## 2. Materials and Methods

The protocol for this systematic review was developed and registered on the Open Science Platform https://osf.io/9kzvx/ (accessed on 11 May 2023). The conduct of this systematic review followed the preferred reporting items for systematic reviews and meta-analyses (PRISMA) statement [23].

Studies were eligible for inclusion in the review if they addressed dietary intake in MAs. The Australian Masters Games, World Masters Games, World Masters Athletics, and Pan Pacific Games were used for MA age cut-off by sport. Criteria for exclusion were (1) animal studies, (2) non-English papers, (3) not a primary study design (poster, review article, editorial, or nutrition recommendation articles), (4) wrong age, (5) wrong population (retired/former MA if not currently fitting the description of an MA, self-defined professional/elite and Olympic athletes, or athletes with disabilities), (6) non-dietary-related (drug use/doping; nutrition habits/knowledge only), (7) not a full-text article (conference abstracts), (8) age not mentioned, (9) unable to separate by age, (10) inadequate dietary data available (alcohol, hydration status, supplements only, and dietary intervention studies without baseline intake), and (11) diets that failed to capture normal/usual intake (e.g., Ramadan, COVID-19, specific competition diets, eating disorders, rapid weight-loss diets, or studies that exclusively examine pre/post-meal recovery).

A comprehensive search strategy was developed with an academic librarian. Subject headings and keyword search terms were formed for the two domains of “masters athletes” and “dietary intake”. Keywords were combined using the Boolean operator “OR”, and domains were combined with “AND”. Due to the limited research available within the two domains, broad search terms were used to maximise the search results relevant to the population of this study. A literature search was conducted in August 2021 and updated in June 2022. The search was conducted in seven electronic databases: Medline (via Ovid), Embase (via Ovid), CINAHL (via EBSCO), Web of Science, SPORTDiscus (via EBSCO), Scopus, and AUSPORT. In addition, handsearching was carried out to identify key articles that may have been missed during the database search. An example of the search strategy conducted in MEDLINE is provided in Supplementary Material Table S1.

Endnote X9.3.1 citation management software (Thomson Reuters, Philadelphia, PA, USA) was used to download citations and abstracts of studies retrieved from the database search and remove duplicate articles. An online screening and data extraction tool, Covidence (Veritas Health Innovation, Melbourne, Australia), was used to further exclude duplicates and screen articles based on the eligibility criteria. Titles and abstracts were independently reviewed by two reviewers (SG and GS) during the first stage of screening. The full text was retrieved for all eligible articles and screened independently. Discrepancies were resolved by third reviewers (JG and WSS).

Data from eligible studies were independently extracted by two researchers (SG and GS) with discrepancies discussed following extraction and resolved. For RCTs, pre-intervention dietary data were extracted as they were representative of the usual diet. Two longitudinal studies assessed both baseline and follow-up dietary intake [24,25], but only baseline data were included in this review to reflect usual intake. Supplements were included. Energy intake data reported as kilocalories (kcal) were multiplied by 4.184 to convert to kilojoules (kJ) for consistency. Only studies with data reported in the specified units—kilojoules per day (kJ/day), grams per day (g/day), grams per kilogram of body weight per day (g/kg BW/day), percent energy (%E), and milligrams per day (mg/day)—were included in the calculation of each value.

Quality and risk of bias was assessed independently by two reviewers (SG and GS) using the Academy of Nutrition and Dietetics Evidence Analysis Manual Quality Criteria Checklist: Primary Research ratings [26]. Any discrepancies were discussed and resolved with third reviewers (JG and WSS).

The participant and study characteristics and dietary intakes of MAs were summarised in a tabular form. Dietary intake data were grouped into energy, the intake of macronutrients, protein (g; g/kg BW; %E), total fat (g; g/kg BW; %E), carbohydrate (g; g/kg BW; %E), and alcohol (g/day; %E), key micronutrients (mg) for athletes (calcium, magnesium, iron, zinc, and sodium), and food or beverage items/food. These five micronutrients were selected for comparison as they have been associated with chronic disease [27] and/or higher prevalence of inadequate intakes [28,29]. The results for each variable were tabulated in Microsoft Excel, according to age, younger (35–50 years) and older (>50 years), and gender (combined data (male and female), male only, female only, and unable to separate by gender). The cut-off of 50 years was deliberately chosen to match the cut-off at middle age for reported nutrients in the AHS data and the marked change in metabolic conditions that occurs around that age for men and women [8,30,31]. MAs were separated into ‘younger’ and ‘older’ age groups based on the mean age (where provided), or the mean was calculated using the age range provided. For the studies where genders were unable to be separated, the results were only included in the combined data category, which included both males and females. Where a range was reported, the mean was taken. The mean for each variable was calculated and the count depended on the number of studies reporting the variable. For example, energy intake of younger MAs (combined data) included eight studies to calculate the mean, while five studies were used to calculate %E for fat.

MA dietary intake data were compared with the latest 2011–2012 Australian population dietary data from adults aged 31–50 years and over 50 years [6]. Data were averaged across the 51–70 years and 71 years and over age groups to yield a single value for comparison. The percentage difference was calculated for the categories of combined data, males only, and females only using the formula % difference = 100 × (value A − value B)/(value A + value B)/2, where A is the MA data. An arbitrary 10% difference cut-off was chosen to highlight differences between MAs and the general population from the 2011–2012 AHS for discussion.

## 3. Results

### 3.1. Study Selection

**Figure 1:**
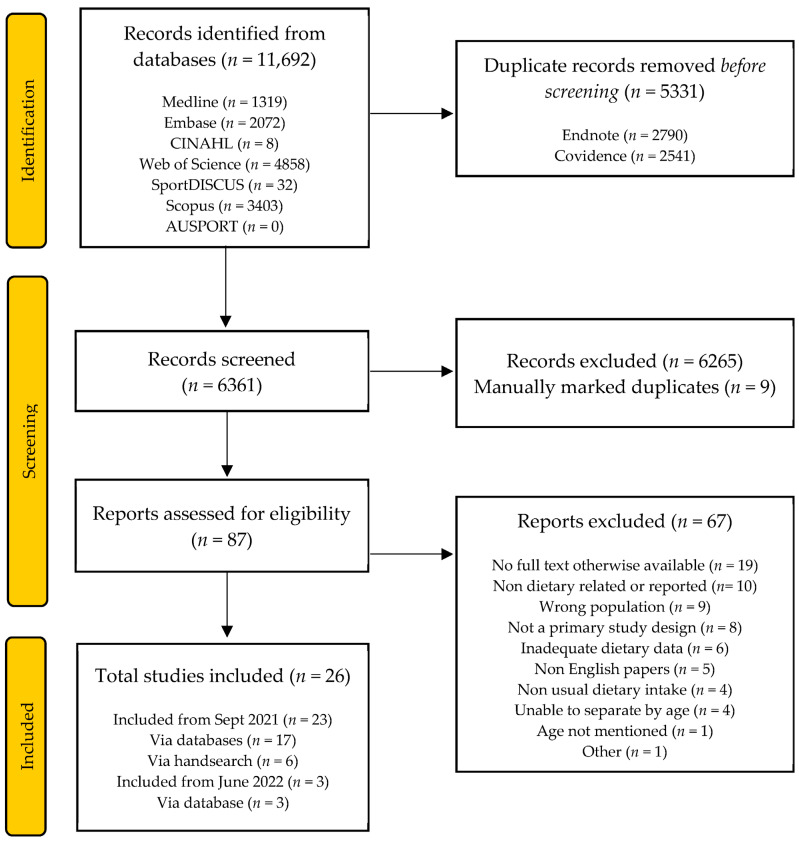
PRISMA flow diagram of record identification and study selection for a systematic review of the dietary intake of masters athletes.

The initial search identified a total of 10,693 articles. Following title and abstract screening and exclusion at the full-text stage, 19 articles remained for inclusion. Handsearching provided an additional six articles. The search was repeated in June 2022 and identified a total of 11,692 articles. Duplicates were removed using automation tools and manually identified during title and abstract screening. After applying the exclusion criteria, three articles remained. In addition to the studies identified from the initial search, a total of 26 articles met the inclusion criteria. This process is summarised in Figure 1.

### 3.2. Participants and Study Characteristics

Participant and study characteristics of the 26 included studies [2,11,12,13,14,15,24,32,33,34,35,36,37,38,39,40,41,42,43,44,45,46,47,48,49,50], including quality ratings, are summarised in Table 1. Of these, seventeen were cross-sectional [2,11,12,13,14,15,32,33,34,37,38,42,43,44,46,47,48], three were RCTs [35,40,41], two longitudinal [24,45], two case studies [39,50], one was a validation study [36], and one a before-and-after study [49]. Three studies were published in the 1980s [11,14,37], two in the 1990s [12,13], five between 2000 and 2010 [2,15,24,32,45], and the remaining published after 2010 [33,34,35,36,38,39,40,41,42,43,44,46,47,48,49]. A total of 2819 participants were included. Twelve studies were male-only [2,12,13,15,37,38,39,40,41,43,45,49], four studies female-only [24,32,35,50], and the remaining nine included both males and females [11,14,33,34,36,42,44,46,47,48]. Eleven studies were conducted across European countries: Italy [33,34,45], the United Kingdom [39,40,41], France [12], the Netherlands [46], Poland [43], Finland [15], and Denmark [38]; eleven studies were conducted in the USA [2,11,13,14,24,32,35,37,47,49,50], two in Canada [36,44], one in South Africa [42]; one included participants from 21 countries [48]. Nineteen studies were on competitive athletes [2,13,15,24,32,33,34,35,36,37,38,39,40,42,43,45,47,49,51], two on recreational athletes [41,44], and five did not report participation level [11,12,14,46,50]. Dietary assessment methods included food records (*n* = 16) [2,11,12,13,14,15,24,32,33,35,39,40,41,42,45,47], food frequency questionnaires (FFQs) (*n* = 8) [24,36,37,38,43,44,46,49], and questionnaires on dietary behaviours [48] and diet history [34].

Results of baseline energy, nutrient, food and beverage items, or food groups are presented in Table 2. Seventeen studies reported energy in kJ/day [2,11,12,13,14,15,32,33,34,35,42,44,45,46,47,49,50]. The range of energy intake in kJ/day was larger for male athletes (range = 6686–14,535 kJ/day) [2,11,12,13,14,15,42,44,45,47,49] compared to female athletes (range = 5073–9983 kJ/day) [11,14,32,35,42,44,47,50]. Eighteen studies reported protein intake in g/day, %E, or g/kg/BW [2,11,12,13,14,15,32,34,35,39,40,41,42,44,45,47,49,50]. Protein intake for males ranged from 57 to 131 g/day [11,12,13,14,44,45,47,49], 13 to 18%E [2,11,13,14,15,39,45,49], and 1.0 to 2.0 g/kg/BW [2,13,14,15,39,40,41,42,47,49]. Protein intake for females ranged from 74 to 104 g/day [11,14,32,35,44,47], 14 to 20%E [11,14,32], and 1.2 to 1.3 g/kg/BW [14,42,47,50]. Eighteen studies reported fat intake in g/day, %E, or g/kg/BW [2,11,12,13,14,15,32,34,35,39,40,41,42,44,45,47,49,50]. Fat intake for males ranged from 51 to 134 g/day [11,12,13,14,44,45,47,49], 22 to 41%E [2,11,13,14,15,39,42,45,49], and 1.0 to 9.0 g/kg BW [2,15,39,40,41,47,49]. Fat intake for females ranged from 61 to 111 g/day [11,14,32,35,44,47], 28 to 41%E [11,14,32,42], and 0.7 to 1.2 g/kg/BW [47,50]. Twenty studies reported carbohydrate intake in g/day, %E, and g/kg/BW [2,11,12,13,14,15,32,34,35,36,39,40,41,42,44,45,47,49,50]. Carbohydrate intake for males ranged from 221 to 350 g/day [11,12,13,14,44,45,47,49], 40 to 61 %E [2,11,13,14,15,39,45,49], and 3.0 to 5.3 g/kg BW [2,15,39,40,41,42,47,49]. Carbohydrate intake for females ranged from 183 to 292 g/day [11,14,32,35,44,47], 40 to 55%E [11,14,32], and 3.5 to 4.1 g/kg BW [42,47,50]. Eight studies reported alcohol consumption in g/day, %E, kJ/day, % consumers, mL, glasses, bottles, or drinks [2,11,13,37,38,44,46,48]. Seven studies reported micronutrients in mg/day [12,14,24,32,40,44,47], including calcium [12,14,24,32,44,47], magnesium [12,14,32], iron [12,14,32,40,44], zinc [14,32,44], and sodium [14,32,44]. Eight studies reported food or beverage items or food groups [14,24,37,38,43,44,46,48]. 

**Figure 2:**
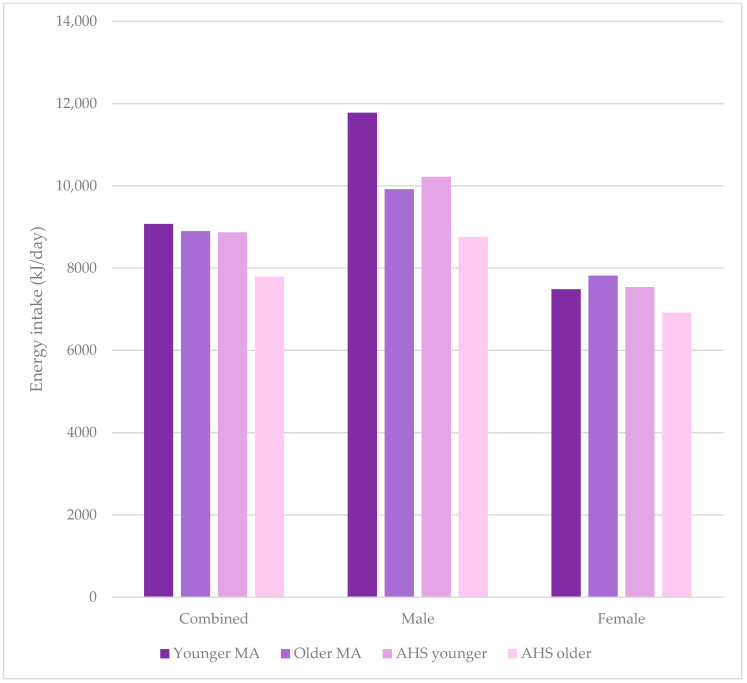
Energy intake of masters athletes compared to 2011–2012 Australian Health Survey data for all people, males, and females, comparing younger masters athletes, older masters athletes, younger people in the Australian Health Survey, and older people in the Australian Health Survey.

**Figure 3:**
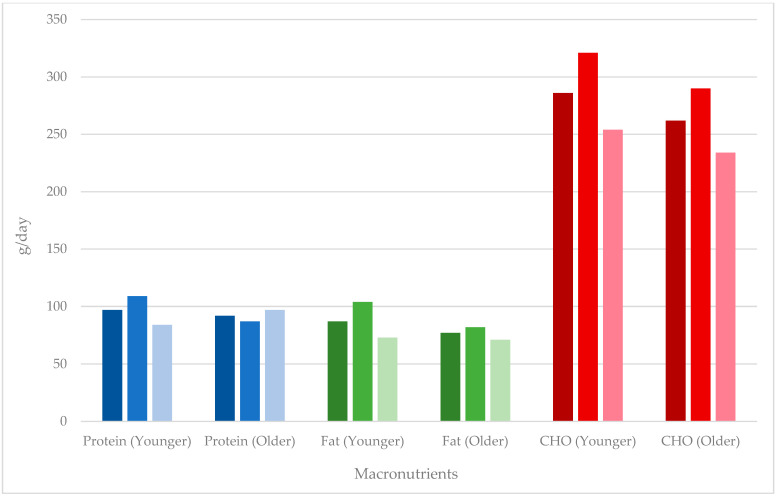
Macronutrient intake of masters athletes. Data presented as combined (males and females), males, and females from left to right (darkest to lightest) for each macronutrient.

Table 3 shows a comparison between the younger and older cohorts for combined, males, and females, with Figure 2 and Figure 3 showing a comparison of energy and macronutrient intake in these cohorts, respectively. Younger male MAs had a higher overall energy intake compared to older male MAs, with that for females and combined younger and older MA groups being equivalent. Apart from alcohol, the greatest %E differences were for fat and protein in females, with younger MAs having a lower %E protein and greater %E fat. The amounts of macronutrients per kg were very similar across groups except for fat, which was greater in older males, pushing up the combined intake for older MAs. Intake of micronutrients was lower for younger MAs (combined) for magnesium, zinc, and iron and for younger female MAs for all micronutrients except sodium. Calcium intake was higher for younger compared with older male MAs, and the opposite was true for females.

Table 4 and Table 5 show the combined averages of nutrients for MAs 35–50 years and >50 years, respectively, with comparison to data from the AHS and percentage differences between the cohorts. There was a greater difference in energy intake for older MAs and younger male MAs compared to the Australian population data. The data indicate that carbohydrate intake is higher for MAs, with carbohydrate g/day and %E being comparatively higher, particularly for older MAs. Calcium, iron, zinc, and sodium intakes were higher in MAs (both younger and older) compared with the population data. Differences were not noted for magnesium in comparisons to population data with younger MAs (combined, male, and female) or older male MAs.

## 4. Discussion

To date, research investigating the usual dietary practices of MAs is limited. Nutrition adequacy in relation to health and sporting requirements is uncertain and complicated by the metabolic challenges that may accompany ageing, the presence of chronic disease and/or risk factors, changing physiological systems, increasing prevalence of supplement use with age, and the need for sport-specific information regarding training load and/or energy expenditure. This is the first systematic review to synthesise the available data and provide a summary of the nutritional intakes of MAs. The findings provide a basis for comparison between MAs and the general population to better understand nutritional adequacy. Higher energy intakes in MAs were generally reported in comparison to the Australian national average intakes, as well as higher micronutrient intakes, and they were more likely to meet national dietary guidelines.

Included studies highlight the heterogeneity of MAs (Table 1). Analysis of MA data across sports showed a wide variation in energy intake, ranging from 5073 kJ/day (one female triathlete) [50] to 14,535 kJ/day (average of nine male triathletes) [42]. While individual (e.g., gender and age), sport (including the primary energy system), and contextual (e.g., goals for adaptation and/or body composition and individual training load) factors [53] should be taken into consideration in assessing adequacy, it is possible that some MAs are not consuming enough to meet their health and sporting requirements, compromising performance, recovery, and desired physiological adaptations.

On the other hand, self-reported dietary intake data have known limitations. Indeed, in the current study, younger female MAs reported a similar energy intake to younger females in the AHS and older female MAs (Figure 2, Table 3 and Table 4), where they would be expected to consume more than both of these groups due to their activity and age. This pattern was not observed for males (Figure 2, Table 3 and Table 4), who followed the expected order. The differences between younger MAs and older MAs, younger MAs versus AHS, and older MAs versus AHS was 17%, 14%, and 12%, respectively. In fact, the energy requirement of women aged 30–60 years with active or moderately active lifestyles is suggested to be >8.9 MJ (≥60 kg body weight) [54], whereas younger MAs had an average of 7485 kJ/day. This is suggestive of underconsumption or underreporting of energy intake. While the systematic review of McKenzie et al. (2021) [55] on sex differences in the accuracy of energy intake assessment (versus doubly labelled water) showed that underreporting across dietary assessment methods was similar in males and females; there have been few studies comparing the reporting of male and female athletes specifically. No known studies investigate the accuracy of reporting of MAs. Alternatively, underconsumption may be part of a state of low energy availability (LEA), where energy intake minus exercise energy expenditure and energy intake (per kg of fat free mass) is suboptimal for health [56]. In this case, the lower energy intake may support exercise (although perhaps not at peak) through “compensatory energetic savings in other processes” but results in maladaptive physiological responses [57]. Logue et al. (2019) [58] reported that 17% of women aged 35–44 across a range of sporting levels in endurance activities were at risk of LEA, supporting this possibility in the younger MAs in the current study.

As expected, younger male MAs reported the highest protein intakes (109 g/day) given their higher energy intake. Older female MAs were found to have higher absolute intakes of protein compared to their younger counterparts (97 g/day vs. 84 g/day) (14% difference; Table 3), suggesting that the larger energy intake reported by older female MAs may have facilitated a higher intake of protein. This finding is reassuring because older people require more protein for the maintenance of muscle mass, good health, and functionality [16]. Additionally, for women, having more skeletal muscle mass may also provide some protection against osteoporosis [59]. Turning to athletic requirements, protein and carbohydrates are generally prescribed on a g/kg basis rather than on percentage of energy, and this may fluctuate daily depending on activity. Protein requirements for athletes are likely to be met with 1.2–2.0 g/kg per day, and requirements for older (nonathletic) individuals are suggested to be ≥1.2 g/kg/day [16]. In the current review, protein intakes relative to body weight in MAs were reported in 12 out of 26 studies and ranged from 1.0 g/kg/day [42] to 2.0 g/kg/day [15], indicating that athletic needs are likely to have been met. However, it has additionally been proposed that active older individuals consume protein doses of at least 30 g (even up to 35–40 g [60]), approximately 3–4 h apart [3] throughout the day and, in particular, following muscle-damaging exercise [60]. Relative to body weight, this would equate to ~1.7 g/kg of body mass (for a 70 kg athlete) [3], higher than most values reported for MAs in the current study. This may indicate that some MAs may need to prioritise greater protein intakes in order to stimulate muscle protein synthesis and minimise lean tissue loss. More targeted guidelines for protein intake in MAs are warranted.

Considering the higher energy intakes in MAs compared to the general population, it was expected that macronutrient intakes in MAs would also reflect this. This was seen with respect to protein, where younger MAs and AHS data showed small percentage differences (5% or less) in absolute intakes. However, greater differences (12–18%) were observed when intakes were expressed as a percentage of total energy intake (Table 4). These findings imply higher total energy intakes for MAs and, consequently, a lower percentage of their intake coming from protein. Protein intakes observed in the general population that appear to match those of younger MAs may be attributed to Australia’s meat-eating culture encouraging greater consumption [61,62], resulting in only minor percentage differences when compared to MAs (Table 4). In the comparisons of MAs and AHS data, the largest percentage difference in absolute intakes (26%) was observed between older females (Table 5).

Carbohydrates are essential to optimise athletic performance. Depleted carbohydrate stores can hinder performance, so it would be desirable for MAs to achieve the recommended daily fuelling requirements. For younger athletes, this ranges between 3 to 5 g/kg/day and 8 to 12 g/kg/day depending on the activities undertaken [53]. In this review, ten studies [2,15,36,39,40,41,42,47,49,50] reported a g/kg amount, and eight [36,39,40,41,42,47,49,50] reported the type of sport, with all of these being endurance activities. For these activities, recommendations would be 5–10 g/kg/day for days with moderate- to high-intensity activity. In most studies, intakes in MAs fell short of these recommendations, with the highest relative intake reported being 5.4 g/kg BW/day for younger male triathlete MAs [36]. Despite this, similarities between younger MAs and older MAs were noted with percentage differences of 10% (or less) (Table 3). Larger differences were noted for male MAs for protein and fat, contributing to the difference in energy intake between these groups (Table 3). Table 4 and Table 5 highlight greater absolute carbohydrate intakes between both younger MAs and older MAs in comparison to age-matched AHS data (22% and 26% differences, respectively), which may result from the larger energy budgets available in MAs.

As seen in Table 3, larger percentage differences were noted in fat intakes between younger MAs and older MAs when expressed relative to body weight; however, the findings are skewed by a single study suggesting intakes of 9.0 g/kg BW/day [2] and, likely, a misreported value. In comparisons to the general population, MAs reported higher intake of fat (Table 4 and Table 5). The largest percentage differences were observed with absolute fat intakes when comparing younger male MAs and older female MAs to age-matched AHS data (19% and 18%, respectively).

Even with greater absolute intakes of carbohydrates and fats demonstrated in MAs, consideration of type and quality is important in the context of chronic disease development. Diets with a lower glycaemic load have demonstrated improved glycaemic control [63], reducing the risk of impaired glucose tolerance or the development of insulin resistance, both of which are major risk factors for type 2 diabetes. Such diets are typically characterised by higher intakes of fruits and vegetables, corresponding with increased total dietary fibre, and these positive dietary patterns are evident in MAs [36,46,48]. Similarly, the types of fat consumed differ in their metabolic impacts. Saturated and trans-fatty acids can contribute to unfavourable blood lipid profiles, increasing the risk of coronary heart disease (CHD), while unsaturated fatty acids aid in lowering cholesterol and contribute to a reduced risk of CHD [17]. One study reported a lower consumption of high saturated-fat foods compared to inactive controls in younger MAs [37]. It is also possible that higher fat intakes in MAs may reflect the popularity of low-carbohydrate, high-fat diets claimed to induce ‘glycogen-sparing’ to enhance performance [64]. It should be noted, though, that these diets have been shown to impair carbohydrate utilisation and the ability to sustain high-intensity performance [65]. The studies within this review do not report on the specific types consumed or dietary patterns, therefore precluding such conclusions from being drawn.

As energy needs often reduce with ageing [3], greater intakes provide MAs with a larger ‘budget’ with which to achieve nutritional adequacy. Studies show that MAs may adopt healthier lifestyles and have more nutritionally complete, higher-energy diets compared to their sedentary counterparts [11,12,13,14,15]. This may further contribute to a reduced risk of lean tissue loss, sarcopenia, and functional decline in combination with the benefits of ongoing exercise [3,10]. This is in contrast to the prevalence of poor dietary habits observed in older Australians, with adults aged 51 years and older found to consume at least 30% of energy intake from discretionary sources [6]. 

Eight [12,14,24,32,40,44,47,50] out of 26 studies included the five key micronutrients selected for comparison in the data analysis, however, were not comprehensively reported across these. The interpretation of the findings is therefore unlikely to be representative across all MAs or of the usual dietary intakes. It is interesting to note that some of the largest percentage differences in the present review were observed in the micronutrient comparisons, with a difference of 125% between older female MAs’ iron intakes and those of their general population counterparts (Table 4). This may be explained by the known skewed distribution of iron intakes [66] and supplement use among MAs [32,67]; however, the use of supplementation to achieve recommended intakes should be cautioned against. Female runners included in a similar study [24] showed higher intakes of calcium from supplementation compared to dietary calcium, suggesting that diet alone was inadequate. The importance of dietary calcium is reflected in the changing nutrient recommendations across life stages to protect and maintain bone health. There is also some emerging evidence to suggest an association between calcium supplementation and an increased risk of cardiovascular disease [68]. In the present review, sodium was only reported in three studies [14,32,44], where MAs demonstrated higher intakes compared to the general population. Reducing sodium for risk-factor management is important with ageing, with the prevalence of hypertension reported to be three times higher among Australians aged 45–54 years than 35–44 years [25]. While hypertension prevalence is lower among MAs [9], intakes above the suggested dietary target (SDT) (2000 mg/day) may have negative impacts on the future risk or current management of the condition. Higher intakes may relate to rehydration and electrolyte replacement strategies [53]; however, this cannot be confirmed given the lack of detail on supplementation or exercise-specific nutrition strategies.

The strengths of this review include the systematic nature of the search and the extensiveness of the data extraction. The majority of the literature was published within recent years, reflecting contemporary consumption patterns [69]. Additionally, both genders were represented, providing a more inclusive dietary assessment. However, this review has a few limitations. Differences between MAs and AHS data may have been influenced by differences in the dietary methodologies used. The majority (16 out of 26) of MA studies used FR and some used FFQ (8 out of 26 studies), while the AHS data were collected with interviewer-administered 24 h recalls [6]. However, in a systematic review of the validity of dietary assessment methods compared to doubly labelled water, the majority of studies reported significant underreporting, and FR, FFQ, and 24 h recall produced underestimations to a similar degree (11–41%, 5–42%, and 8–30%, respectively) [70]. Additionally, there was inconsistency in the data reported across the studies. For example, variation in units of measurement provided some challenges in analysis; however, the calculation of combined average intake values enabled some degree of comparison. An average value was also calculated for Australian population data across the 51–70 and 71 and over age groups to allow for comparisons with older MAs because studies did not separate MA results by age and age-matched comparisons were not possible. For MA data, the calculation of male- or female-only results excluded data from mixed-cohort studies, where data for each gender could not be separated out, which reduced their pool size. Endurance sports were disproportionately represented among the studies in this review, and comparisons between competitive and noncompetitive MAs could not be undertaken as many studies failed to specify the level of competition for their athletes. As masters-level competitions often do not have qualification requirements, ‘competitive’ MAs may not be substantially different from recreational MAs, and they may be similarly unaware of sport-specific dietary practices.

## 5. Conclusions

The available data indicate that, for the most part, MAs appear to have higher absolute intakes of energy, macronutrients, and included micronutrients compared to the general Australian population. They also compared more favourably, with the exception of sodium, with the Australian dietary recommendations for key nutrients measured. In conjunction with exercise, MA dietary practices may attenuate age-associated physiological declines, with potential improvements in the chronic disease risk profile. More comprehensive assessments of dietary intake to ascertain diet quality in relation to health, with larger sample sizes and addressing various sporting disciplines (endurance-based, power-based, and mixed sports and reported as such) would be beneficial. Studies should adopt a longitudinal approach across all phases of competition and training to account for dietary variations, with further exploration into the rationale behind their dietary practices. These insights will inform future research, sports nutrition guidelines for competitive MAs, and nutrition strategies for healthy ageing among the general population.

## Figures and Tables

**Table 1:** Participant and study characteristics of the included studies.

First Author, Year, Reference	Study Type	Aim	Sport *	Country	Level **	Age (Years) ***	Number (n)Gender (M/F)	Dietary Assessment Method	Quality Rating (ADA) [26]	Funding
Beshgetoor et al., 2000 [24]	Longitudinal (Baseline and 18MO for Ca intake)	Determine the effect of sport-specific training and Ca intake on BMD	Cycling (*n* = 12) Running (*n* = 9)	USA	Competitive	49.6 ± 7.9 Cyclists = 48.2 ± 8.4 Runners = 50.9 ± 7.5	*n* = 21 21 F	4D FR 3 weekdays/1 weekend Validated FFQ specific for Ca	(+)	NR
Beshgetoor & Nichols, 2003 [32]	Cross-sectional	Compare the dietary intakes of supplementing athletes with nonsupplementing athletes	Cycling Running	USA	Competitive	50.4 Supplementing = 52.5 ± 2.0 Non-supplementing = 48.4 ± 2.4	*n* = 25 25 F Supplementing (*n* = 16) Non-supplementing (*n* = 9)	4D FR 3 weekdays/1 weekend	(+)	NR
Blair et al., 1981 [11]	Cross-sectional	Compare nutrient intake in regular runners with sedentary individuals of the same age and sex	Long distance running (*n* = 61)	USA	NR	35–59	*n* = 61 (34 M, 27 F)	3D FR Most diet records were obtained on running days	(Ø)	Supported in part by LRC Contract, NIH grant, and a grant from Best Foods, a Division of CPC International
Chatard et al., 1998 [12]	Cross-sectional	Examine the nutritional status of sportsmen and to evaluate its relationship to physical fitness	Cycling Running Swimming Tennis Walking #	France	NR	63 ± 4.5	*n* = 18 18 M	2 × 3D weighed FR, with a 6W interval 3 consecutive weekdays (Tuesday–Thursday)	(+)	NR
Condello et al., 2019 [33]	Cross-sectional	Investigate the mediating effects of total energy expenditure and intake, body mass, and body image dissatisfaction on the relationship between age and perception of health and quality of life	Senior athletes *n* = 42	Italy	Competitive (National/International)	55−84	*n* = 42 M & F !	7D FR	(+)	Ministero dell’Istruzione, dell’Università e della Ricerca
Croteau et al., 2021 [51]	Cross-sectional	Describe and compare health status, lifestyle behaviours, and well-being of athletes from three world regions competing in the 2018 Masters Field Hockey World Cup	Hockey	21 countries clustered into North America (*n* = 138), Europe (*n* = 273), Asia/Pacific (*n* = 54)	Competitive (Masters Field Hockey and Grand Masters World Cups)	35–76	*n* = 465 180 M; 284 F; 1 O	Questionnaire on lifestyle variables related to dietary behaviours, alcohol use, sleep, sitting time, and physical activity	(+)	NR
Di Girolamo et al., 2017 [34]	Cross-sectional	Test the hypothesis that protein intake level is associated with muscle strength in elderly elite athletes	Cycling Running Pentathlon Fencing Weightlifting	Italy	Competitive (European Master Games 2011)	65–81	*n* = 50 (38 M, 12 F) § LPI *n* = 25 (19 M, 7 F) § HPI *n* = 25 (18 M, 6 F) §	DHx (over 1 W period)	(Ø)	A grant from the Italian Ministry of Education, University and Research
Glenn et al., 2015 [35]	RCT	Examine the longitudinal effects of beta-alanine on time to exhaustion, total work completed, and lactate clearance in female master athlete cyclists	Cycling	USA	Competitive	Beta-alanine = 54 ± 2 Placebo = 53 ± 1	*n* = 22 22 F Beta-alanine *n* = 11 Placebo *n* = 11	3D FR 2 non-consecutive weekdays and 1 weekend	(+)	Powder City donated the beta-alanine
Hallfrisch et al., 1994 [13]	Cross-sectional	Compare the fitness, body composition, and diet intakes of older endurance-trained men and a group of healthy, but sedentary men of similar age who were matched for BMI	Running (*n* = 11) Bicycling (*n* = 5) Racquetball (*n* = 1) Swimming (*n* = 5) Race walking (*n* = 8) Weightlifting (n = 1) #	USA	Competitive (Senior Olympics and/or competed at national/local level)	58–75 Athletes = 66.6 ± 1.3	*n* = 16 16 M	7D FR (athletes and controls during a normal training week)	(+)	NR
Harrison et al., 2018 [36]	Validation	Develop and validate a rapid and easy to use dietary screener to identify athletes who do and do not achieve a CHO intake >6 g/kg BW in the context of endurance sports	Ironman triathlon Ironman 70.3 triathlon Winter pentathlon (tandem or solo) Winter triathlon	Canada	Competitive	VALID = 37.1 ± 11.3	*n* = 175 (111 M, 64 F)	Validated web-based FFQ—136 questions Food intake 1MO prior	(Ø)	A grant from Canadian Institutes of Health Research
Hartung et al., 1980 [37]	Cross-sectional	Investigate relationship between diet and plasma lipids and lipoprotein levels in middle-aged marathon runners, joggers, and inactive men	Marathon running (*n* = 59) Jogging (*n* = 85)	USA	Competitive (marathon runners) NR (joggers)	35–66 Marathon = 44.4 ± 6.8 Joggers = 46.8 ± 7.6	*n* = 144 144 M	FFQ—25 foods or groups of foods Asked servings (D, W, MO, Y)	(+)	NR
Hjerrild et al., 2019 [38]	Cross-sectional	Investigate the effect of regular long-term exercise and diet on skin autofluorescence as a measure of glycation and on Achilles tendon structure	Athletics (*n* = 167) Running (*n* = 15)	Athletes of 24 different nationalities competing at the 2017 European Masters Athletics Championships Stadia/Denmark	Competitive (2017 European Masters Athletics Championships Stadia)	Athletes = 57 ± 15	*n* = 182 182 M	FFQ (over past 3MO in fixed ranges D, W)	(+)	Lundbeckfonden
Louis et al., 2020 [39]	Case study	Evaluate the feasibility and benefits that evidence-based nutritional and training recommendations could have on the time course of reconditioning and retraining following hip arthroplasty in an endurance master triathlete	Triathlon	UK	Competitive (70.3 Ironman age group World Championship)	52	*n* = 1 1 M	7D FR—consecutive days Remote food photographic method	(Ø)	No
Mehta et al., 2019 [50]	Case study	Examine the post-workout effects of ingesting beef or whey protein extracts or CHO in female endurance athletes during a 10 W endurance training program	Triathlon	UK	NR	40–55	*n* = 6 6 F	NR	(Ø)	Crown Sports Nutrition and the University of Greenwich
Naclerio et al., 2017 [40]	RCT	Compare the effect of ingesting hydrolysed beef protein, whey protein, and CHO on performance, body composition, muscular thickness, and blood indices of health, including ferritin concentrations, following a 10 W intervention program	Triathlon	UK	Competitive	35–60 Beef = 47.0 ± 8.9 Whey = 45.3 ± 8.9 CHO = 46.2 ± 7.0	*n* = 24 24 M Beef *n* = 8 Whey *n* = 8 CHO *n* = 8	3D FR 2 weekday/1 weekend Baseline and during the last week of intervention	(+)	Crown Sports Nutrition and the University of Greenwich
Naclerio et al., 2019 [41]	RCT	Examine the long-term effects of ingesting hydrolysed beef protein versus carbohydrate on indirect markers of immunity during 10 weeks of endurance training in master aged triathletes	Triathlon	UK	Recreational	35–60 PRO = 48 ± 8 CHO = 46 ± 6.9	*n* = 16 16 M PRO *n* = 8 CHO *n* = 8	3D FR 2 weekday/1 weekend Baseline and during the last week of intervention	(+)	Crown Sports Nutrition and the University of Greenwich
Nieman et al., 1989 [14]	Cross-sectional	Compare food records from marathon runners to various standards of dietary quality	Marathon running	USA	NR	M = 40.1 ± 0.6 F = 37.8 ± 1.2	*n* = 347 (291 M, 56 F)	3D FR—consecutive days 2 weekday/1 weekend (Sunday-Tuesday)	(+)	NR
Potgieter et al., 2011 [42]	Cross-sectional	Determine body composition, dietary intake and supplement use among Olympic and Ironman distance triathletes residing in the Western Cape	Triathlon	South Africa	Competitive (Olympic/Ironman distance events)	M = 37.9 ± 6.8 F = 37.5 ± 9.6	*n* = 26 (total study) *n* = 18 (reported dietary data) (9 M, 9 F)	3D FR 2 weekday/1 weekend An additional questionnaire was completed for supplement use	(Ø)	NR
Ratajczak et al., 2021 [43]	Cross-sectional	Evaluate diet quality and its determinants among ageing masters athletes	Athletics	Poland	Competitive (8th World Masters Indoor Athletics Championship)	36–65 Poland = 50.5 ± 8.3 France = 51.1 ± 9.6 Great Britain = 50.5 ± 8.7	*n* = 86 86 M	FFQ (Dietary Habits and Nutrition Beliefs Questionnaire)	(Ø)	No external funding
Sallinen et al., 2008 [15]	Cross-sectional	Compare muscle strength and thickness, body composition and dietary intake between master strength athletes and controls	Shot-put Discus Hammer throw	Finland	Competitive (national)	Middle-aged athletes = 52.1 ± 4.7 Older athletes = 71.8 ± 3.8	*n* = 17 17 M Middle-aged athletes *n* = 9 Older athletes *n* = 8	4D FR 3 weekday/1 weekend	(Ø)	Grant from the Ministry of Education and a contribution of Peurunka-Medical Rehabilitation Center, Finland
Shaw et al., 2021 [44]	Cross-sectional	Investigate how the COVID-19 pandemic impacted the diet, training and fitness of masters-level cyclists	Cycling	Canada	Recreational (as defined by Priego Quesada et al., 2018 [52])	46 ± 10	*n* = 32 (12 M, 20 F)	FFQ (adapted for use in the Canadian population) Typical diet prior and during the COVID-19 pandemic	(Ø)	No financial support
Sullo et al., 2004 [45]	Longitudinal	Investigate the changes in body composition and aerobic power in a group of older athletes who practiced competitive sport for two consecutive years comparing them with a control group of subjects with similar characteristics who practiced moderate physical activity	Endurance sports (NS) (*n* = 20)	Italy	Competitive	65 ± 2.6	*n* = 20 20 M	7D weighed FR—consecutive days Beginning of the study and for two consecutive years	(Ø)	NR
Van der Avoort et al., 2021 [46]	Cross-sectional	Establish if there is an association between habitual PA and intake of nutrient-dense foods	NS	Netherlands	NR	(PA quintiles) Q4 = 62 ± 8 Q5 = 64 ± 8	*n* = 987 Q4 *n* = 494 (274 M, 220 F) Q5 *n* = 493 (304 M, 189 F)	Online validated 180-item semi-quantitative FFQ	(+)	Part of the EAT2MOVE project and supported by a grant from the Province of Gelderland
Van Pelt et al., 2001 [2]	Cross-sectional	Test the hypotheses that (1) RMR is lower with age in men who regularly perform endurance exercise, and (2) this age-related decline in RMR is related to declines in exercise volume and/or estimated energy intake	Running Triathlon (Active (*n* = 32))	USA	Competitive	63 ± 1	*n* = 26 ^ 26 M	4D weighed or measured FR—consecutive days 3 weekday/1 weekend	(+)	Public Health Services Research Grant 5 01 RR-00051 from the Division of Research Resources
Viner et al., 2015 [47]	Cross-sectional	(1) Examine EA of adult male and female competitive cyclists across the cycling training and competition season, (2) analyse eating behaviours that may contribute to LEA, and (3) compare EA of male versus female cyclists, and endurance road cyclists versus mountain bike cyclists	Cycling Road (*n* = 5) Mountain (*n* = 5)	USA	Competitive	M = 42.0 ± 7.7 F = 38.4 ± 10.3	*n* = 10 (6 M, 4 F)	3D FR per MO through one cycling season (alternating days each MO to represent all days of the week) Encouraged to use a scale or household measures to quantify food intake	(+)	NR
Waldman et al., 2022 [49]	Before and after	Examine the effects of a 21D low CHO, high fat diet on 30 inflammatory markers in endurance-trained, middle-aged men, before and after exposure to exercise and heat stressors	Triathlon	USA	Competitive (recreational level)	39.5 ± 9.9	*n* = 8 8 M	Dietary questionnaire with a list of common CHO rich food and beverages	(+)	NR

BMD = bone mineral density; BMI = body mass index; BW = body weight; Ca = calcium; CHO = carbohydrate; D = day; DHx = diet history; EA = energy availability; F = females; FFQ = food frequency questionnaire; FR = food record; HPI = high protein intake; LEA = low energy availability; LPI = low protein intake; M = males; MO = month; NR = not reported; NS = not specified; O = other; PA = physical activity; PRO = protein; RCT = randomised trial; RMR = resting metabolic rate; USA = United States of America; VALID = sample of non-elite endurance athletes used to validate the screener; W = week; Y = year; * subject numbers included where available; ** noted as competitive or recreational if explicitly stated; otherwise documented as NR (not reported); *** age presented as mean ± SD or range, # several men regularly participated in more than one sport; § number of males, females, and total number of participants in each dietary group is inconsistent with the overall numbers reported for males, females and each dietary group; Academy of Nutrition and Dietetics Evidence Analysis Manual Quality Criteria Checklist quality rating symbols: neutral (Ø), positive (+); ! combined total number of subjects provided, number of male and female subjects not specified; ^ active with plausible dietary data.

**Table 2:** Energy, nutrient, food or food group intake of masters athletes.

First Author, Year, Reference	Study Subgroup	Energy (kJ/Day) Mean ± SD	Protein Mean ± SD	Fat Mean ± SD	Carbohydrate Mean ± SD	Alcohol Mean ± SD	Micronutrients (mg/day)	Food or Beverage Items/Food Groups
Beshgetoor et al., 2000 [24]	Cyclists, Female	NR	NR	NR	NR	NR	Ca ! Baseline = 984 ± 583	No significant differences in frequency of dairy intake between the three groups (cyclists, runners and controls). On average all three groups consumed dairy products >1 per week, but not everyday
	Runners, Female	NR	NR	NR	NR	NR	Ca ! Baseline = 598 ± 457	NR
Beshgetoor & Nichols, 2003 [32]	Supplementing athletes, Female	8699 ± 2628 †	104 ± 75 g 20%E	65 ± 39 g 28%E	269 ± 112 g 52%E	NR	Ca = 1708 ± 127 Mg = 601 ± 58 Fe = 43 ± 8 Zn = 21 ± 2 Na = 2806 ± 980	NR
	Nonsupplementing athletes, Female	8372 ± 1820 †	84 ± 35 g 17%E	61 ± 22 g 28%E	277 ± 43 g 55%E	NR	Ca = 791 ±174 Mg = 366 ± 45 Fe = 25 ± 9 Zn = 13 ± 4 Na = 2897 ± 1243	NR
Blair et al., 1981 [11]	Runners, Male	12380 †	102 ± 30 g 14 ± 3%E	134 ± 39 g 41 ± 7%E	295 ± 87 g 40 ± 8%E	Average 23 g/d 6%E 83% consumers	NR	NR
	Runners, Female	9983 †	82 ± 20 g 14 ± 2%E	111 ± 44 g 41 ± 6%E	234 ± 69 g 40 ± 7%E	16 g/d 5%E 74% consumers	NR	NR
Chatard et al., 1998 [12]	Sportsmen, Male	11549 ± 1923	102 ± 12 g/d	107 ± 18 g	338 ± 94 g/d	NR	Ca = 960 ± 304 Mg = 353 ± 79 Fe = 18 ± 4	NR
Condello et al., 2019 [33]	Senior athletes (55–84 years), Male/Female	8208 ± 1540 †	NR	NR	NR	NR	NR	NR
Croteau et al., 2021 [51]	Hockey, North America	NR	NR	NR	NR	Alcoholic beverages in a week n (%) Do not consume: 29 (21) Less than 1: 22 (15.9) 1 to 2: 29 (21) 3 to 4: 27 (20) 5 to 6: 16 (12) 7 or more: 15 (11)	NR	Serves of Fruit n (%) Do not eat: 1 (1) One: 41 (30) Two: 56 (41) Three: 30 (22) Four or more: 10 (7) Serves of Vegetables *n* (%) Do not eat: 1 (1) One: 19 (14) Two: 53 (38) Three: 31 (23) Four or more: 18 (13) SSB consumed each day *n* (%) Do not consume: 57 (41) Less than one: 31 (23) One: 32 (23) Two: 12 (9) Three: 4 (3) Four or more: 2 (1)
	Hockey, Europe	NR	NR	NR	NR	Alcoholic beverages in a week *n* (%) Do not consume: 43 (16) Less than 1: 42 (15) 1 to 2: 60 (22) 3 to 4: 51 (19) 5 to 6: 39 (14) 7 or more: 38 (14)	NR	Servings of Fruit *n* (%) Do not eat: 4 (2) One: 70 (26) Two: 92 (34) Three: 59 (22) Four or more: 26 (10) Servings of Vegetables *n* (%) Do not eat: 7 (3) One: 87 (32) Two: 73 (27) Three: 49 (18) Four or more: 57 (21) SSB consumed each day *n* (%) Do not consume: 101 (37) Less than one: 61 (22) One: 50 (18) Two: 34 (13) Three: 12 (4) Four or more: 15 (6)
	Hockey, Asia/Pacific	NR	NR	NR	NR	Alcoholic beverages in a week *n* (%) Do not consume: 14 (25.9) Less than 1: 7 (13) 1 to 2: 11 (20) 3 to 4: 8 (15) 5 to 6: 5 (9) 7 or more: 9 (17)	NR	Servings of Fruit *n* (%) Do not eat: 2 (4) One: 23 (46) Two: 16 (30) Three: 10 (19) Four or more: 3 (6) Servings of Vegetables *n* (%) Do not eat: 0 (0) One: 8 (15) Two: 10 (19) Three: 19 (35) Four or more: 10 (19) SSB consumed each day *n* (%) Do not consume: 23 (43) Less than one: 14 (26) One: 7 (13) Two: 3 (6) Three: 4 (7) Four or more: 3 (6)
Di Girolamo et al., 2017 [34]	Elite senior athletes, low PRO intake, Male/Female	8941 (7514–10021) †,^$^	1.2 (1.1–1.3) g/kg BW ^$^ 16 (15–19)%E ^$^	26 (22–30)%E ^$^	51(48–55)%E ^$^	NR	NR	NR
	Elite senior athletes, high PRO intake, Male/Female	8485 (7268- 9820) †,^$^	1.3 (1.2–1.6) g/kg BW ^$^ 23 (20–27)%E	22 (20–24)%E	49(44–53)%E	NR	NR	NR
Glenn et al., 2015 [35]	Cyclists, beta-alanine, Female	7540 ± 2126 †	90 ± 27 g ^#^	69 ± 22 g ^#^	183 ± 62 g ^#^	NR	NR	NR
	Cyclists, placebo, Female	9162 ± 1997 †	97 ± 27 g ^#^	80 ± 25 ^#^	249 ± 68 g ^#^	NR	NR	NR
Hallfrisch et al., 1994 [13]	Endurance-trained, Male	10297 ± 490 †	103 ± 7 g ^#^ 17 ± 1%E 1.5 ± 0.1 g/kg BW ^#^	85 ± 8 g ^#^ 31 ± 2%E ^#^	312 ± 17 g ^#^ 50 ± 2%E ^#^	EtOH: 9 ± 2 g ^#^ 2 ± 1%E	NR	NR
Harrison et al., 2018 [36]	Endurance athletes, Male/Female	NR	NR	NR	5.4 ± 2.5 g/kg BW	NR	NR	NR
Hartung et al., 1980 [37]	Joggers, Male	NR	NR	NR	NR	Vodka, rum, and whiskey (45 mL): 3.3 ± 5.2	NR	Significant dietary differences between joggers and inactive subjects for the number of portions per week for beef, veal, and pork, sausages, bacon, and sugar, jam, jelly and honey. Beef, veal, and pork (85 g): 5.2 ± 4.1 Sausages (57 g): 0.5 ± 0.8 Bacon (17 g): 1.6 ± 3.3 Cottage cheese (56 g): 1.0 ± 1.6 Sugar, jam, jelly, honey (4 g): 4.8 ± 7.3
	Marathon Runners, Male	NR	NR	NR	NR	Vodka, rum, and whiskey (45 mL): 1.20 ± 2.81	NR	Significant dietary differences between marathon runners and inactive subjects for the number of portions per week for beef, veal, and pork, sausages, bacon, and cottage cheese. Beef, veal, and pork (85 g): 5.6 ± 4.9 Sausages (57 g): 0.5 ± 0.7 Bacon (17 g): 1.4 ± 2.2 Cottage cheese (56 g): 1.6 ± 3.4 Sugar, jam, jelly, honey (4 g): 6.9 ± 10.3
Hjerrild et al., 2019 [38]	Athletes, Male	NR	NR	NR	NR	Mean weekly dietary intake Wine (glasses) 4.2 ± 8.0 Beer (bottles) 2.6 ± 5.8 Liquors/spirits (drinks) 0.4 ± 2.2	NR	Mean weekly dietary intake. Fruit (pieces): 15.3 ± 9.8 Vegetables (100 g portions): 13.5 ± 8.7 Fish (100 g portions): 2.1 ± 3.4 Rye or wholegrain bread (slices): 16 ± 12 Oat or wholegrain cereals (1 dL servings): 5.7 ± 5.9 “Western” diet currently (%):28 ± 21 “Western” diet prior to 18 years (%): 44 ± 25 Coffee (cups/week): 16 ± 12
Louis et al., 2020 [39]	Triathlete, Male	NR	1.7 ± 0.5 g/kg BW 16 ± 3%E	2.0 ± 0.8 g/kg BW 40 ± 9%E	4.5 ± 0.8 g/kg BW 43 ± 7%E	NR	NR	NR
Mehta et al., 2019 [50]	Triathlete, Beef, Female	5073 †	1.3 g/kg/BW	1.1 g/kg BW	3.5 g/kg BW	NR	Fe: 11 Non-Heme: 8 Heme: 3	NR
	Triathlete, Beef, Female	6286 †	1.3/kg/BW	0.9 g/kg BW	4.0 g/kg BW	NR	Fe: 13 Non-Heme: 10 Heme: 3	NR
	Triathlete, Whey, Female	6317 †	1.3/kg/BW	0.7 g/kg BW	3.9 g/kg BW	NR	Fe: 12 Non-Heme: 8 Heme: 3	NR
	Triathlete, Whey, Female	6317 †	1.3/kg/BW	1.2 g/kg BW	4.1 g/kg BW	NR	Fe: 10 Non-Heme: 7 Heme: 3	NR
	Triathlete, Carbohydrate, Female	5483 †	1.3/kg/BW	0.8 g/kg BW	4.0 g/kg BW	NR	Fe: 7 Non-Heme: 6 Heme: 1	NR
	Triathlete, Carbohydrate, Female	6489 †	1.3/kg/BW	1.1 g/kg BW	4.1 g/kg BW	NR	Fe: 12 Non-Heme: 9 Heme: 3	NR
Naclerio et al., 2017 [40]	Triathletes, beef supplementation, Male	NR	1.3 ± 0.3g/kg BW	1.1 ± 0.3 g/kg BW	3.4 ± 1.2 g/kg BW	NR	Fe: 12 ± 4	NR
	Triathletes, whey supplementation, Male	NR	1.5 ± 0.6 g/kg BW	1.4 ± 0.4 g/kg BW	3.5 ± 1.6 g/kg BW	NR	Fe: 15 ± 9	NR
	Triathletes, CHO, Male	NR	1.3 ± 0.2 g/kg BW	1.4 ± 0.8 g/kg BW	3.0 ± 1.2 g/kg BW	NR	Fe: 14 ± 3	NR
Naclerio et al., 2019 [41]	Triathletes, PRO group, Male	NR	1.3 ± 0.3 g/kg BW	1.1 ± 0.3 g/kg BW	3.4 ± 1.2 g/kg BW	NR	NR	NR
	Triathletes, CHO group, Male	NR	1.4 ± 0.2 g/kg BW	1.4 ± 0.8 g/kg BW	3.0 ± 1.2 g/kg BW	NR	NR	NR
Nieman et al., 1989 [14]	Marathon Runners, Male	10,569 ± 180 †	105 ± 2 g ^#^ 1.4 ± 0.0 g/kg BW 17%E	87 ± 2 g ^#^ 31%E	327 ± 7 g ^#^ 52%E	NR	Ca: 1034 ± 28 ^#^ Mg: 386 ± 10 ^#^ Fe: 20 ± 1 ^#^ Zn: 12 ± 0.4 ^#^ Na: 3303 ± 81 ^#^	Runners were asked to estimate the kind of changes made in their diets after they began regular running. More than 75% of the runners reported somewhat or definitely higher intakes of fruits, vegetables, whole grains, poultry, and fish and lower intakes of red meat, eggs, salt, and fats
	Marathon Runners, Female	7819 ± 339 †	74 ± 5 g/d ^#^ 1.3 ± 0.1 g/kg BW 16%E	66 ± 5 g/d ^#^ 32%E	246 ± 11 g/d ^#^ 53%E	NR	Ca: 797 ± 53 ^#^ Mg: 299 ± 16 ^#^ Fe: 14 ± 1 ^#^ Zn: 8 ± 1 ^#^ Na: 2583 ± 149 ^#^	NR
Potgieter et al., 2011 [42]	Triathletes, Male	14,535 ± 4510	2.0 ± 0.5 g/kg BW	35.0 ± 10%E	5.3 ± 1.9 g/kg BW	NR	NR	NR
	Triathletes, Female	9004 ± 369	1.2 ± 0.2 g/kg BW	30.0 ± 6.0%E	3.5 ± 1.0 g/kg BW	NR	NS ^	NR
Ratajczak et al., 2021 [43]	Athletics, Poland, Male	NR	NR	NR	NR	NR	NR	Median values Diet quality 25.5 Median consumption frequency Number of meals daily 4.0 Wholemeal bread 0.1 Grains and groats 0.1 Milk 0.5 Fermented milk beverages 0.5 Curd 0.1 White meat 0.5 Fish 0.1 Legumes 0.1 Fruits 1.0 Vegetables 1.0
	Athletics, France, Male	NR	NR	NR	NR	NR	NR	Median values Diet quality 29.8 Median consumption frequency Number of meals daily 4.0 Wholemeal bread 0.5 Grains and groats 0.5 Milk 0.1 Fermented milk beverages 0.3 Curd 0.3 White meat 0.5 Fish 0.5 Legumes 0.1 Fruits 2.0 Vegetables 2.0
	Athletics, Great Britain, Male	NR	NR	NR	NR	NR	NR	Median values Diet quality 31.0 Median consumption frequency Number of meals daily 3.0 Wholemeal bread 0.5 Grains and groats 0.1 Milk 0.1 Fermented milk beverages 0.5 Curd 0.1 White meat 0.5 Fish 0.1 Legumes 0.1 Fruits 1.0 Vegetables 2.0
Sallinen et al., 2008 [15]	Middle-aged Athletes, Male	11,000 ± 1300	1.2 ± 0.3 g/kg BW 18 ± 2%E	1.1 ± 0.3 g/kg BW 36 ± 5%E	3.0 ± 0.8 g/kg BW 43 ± 6%E	NR	NR	NR
	Older Athletes, Male	9300 ± 1200	1.0 ± 0.3 g/kg BW 16 ± 4%E	1.0 ± 0.3 g/kg BW 33 ± 5%E	3.2 ± 0.6 g/kg BW 51 ± 7%E	NR	NR	NR
Shaw et al., 2021 [44]	Cyclists, Male	11,728 ± 2736 †	131 ± 51 g	105 ± 35 g	350 ± 91 g	3.2 ± 4.6 g	Ca: 1539 ± 778 Fe: 24 ± 7 Zn: 21 ± 16 Na: 3831 ± 1209	Caffeine: 155 ± 127 mg
	Cyclists, Female	9021 ± 2410 †	104 ± 31 g	68 ± 22 g	292 ± 95 g	4.0 ± 5.5 g	Ca: 1815 ± 862 Fe: 33 ± 19 Zn: 18 ± 9 Na: 3283 ± 966	Caffeine: 302 ± 157 mg
Sullo et al., 2004 [45]	Endurance sports, baseline, Male	6686 ± 523 †	57 ± 6 g ^#^ 15 ± 2%E	54 ± 7 g ^#^ 31±2%E	221 ± 31 g ^#^ 56 ± 1%E	NR	NR	NR
Van der Avoort et al., 2021 [46]	Active, Q4, Male/Female	9297 ± 3075 †	NR	NR	NR	14 ± 14 g	NR	Fruit and vegetable intake Overall: 363 ± 175 g/day Fruit intake: 125 ± 124 g/day (0.8/serves) Vegetable intake: 168 ± 95 g/day (2.2/serves) % meeting guidelines = 38%
	Active, Male, Q4	10,092 ± 3167 †	NR	NR	NR	NR	NR	NR
	Active, Female, Q4	8301 ± 2636 †	NR	NR	NR	NR	NR	NR
	Active, Q5, Male/Female	9029 ± 2833 †	NR	NR	NR	13 ± 13 g	NR	Fruit and vegetable intake Overall: 386 ± 213 g/day Fruit intake: 210 ± 142 g/day (1.4 serves) Vegetable intake: 177 ± 121 g/day (2.4/serves) % meeting guidelines 41%
	Active, Male, Q5	10,096 ± 2996 †	NR	NR	NR	NR	NR	NR
	Active, Female, Q5	8427 ± 2807 †	NR	NR	NR	NR	NR	NR
Van Pelt et al., 2001 [2]	Physically active, Male	10,326 ± 377 †	1.2 ± 0.1 g/kg BW ^#^ 13%E	9.0 ± 0.6 g/kg BW ^#^ 27%E	4.7 ± 0.2 g/kg BW ^#^ 58%E	226 ± 54 † (2%) kJ/day	NR	NR
Viner et al., 2015 [47]	Cyclists, Male/Female	8715 ± 649 †	94 ± 29 g 1.4 ± 0.4 g/kg BW	72 ± 19 g 1.1 ± 0.3 g/kg BW	267 ± 84 g 3.9 ± 1.2 g/kg BW	NR	Ca: 1400–1900	NR
	Cyclists, Male	9669 ± 2029 †	106 ± 30 g 1.5 ± 0.4 g/kg BW	78 ± 21 g 1.1 ± 0.3 g/kg BW	296 ± 94 g 4.1 ± 1.3 g/kg BW	NR	NR	NR
	Cyclists, Female	7284 ± 1109 †	77 ± 15 g 1.3 ± 0.4 g/kg BW	61 ± 7 g 1.0 ± 0.3 g/kg BW	223 ± 43 g 3.7 ± 1.2 g/kg BW	NR	NR	NR
Waldman et al., 2022 [49]	Triathlete, Habitual Diet, Male	11,799 ± 3996†	103 g ± 47 g 1.3 ± 0.6 g/kg/BW 15 ± 20%E ^#^	116 ± 29 g 1.4 ± 0.4 g/kg/BW 37 ± 27%E ^#^	336 ± 145 g 4.1 ± 1.8 g/kg/BW 48 ± 61%E ^#^	NR	NR	NR

† Converted kilocalories (kcal) to kilojoule (kJ) using 1 kcal = 4.184 kJ; macronutrient values are presented as mean ± standard deviation g/day, g/kg/BW/day, %E as provided by each respective article unless otherwise specified. Values have been rounded to the nearest decimal point where relevant.; ^$^ data presented as median (interquartile range); ^#^ data presented as mean ± standard error of the mean; ! where studies have included supplementation, nutrient data represents intake from diet alone; ^ data presented as a graph however specific values could not be interpreted directly; BW = body weight; Ca = calcium; EtOH = alcohol; Fe = iron; g = grams; kJ = kilojoules; Mg = magnesium; Na = sodium; NR = not reported; NS = not specified; PRO = protein group; Q = quintile; Zn = zinc; %E = percentage of energy intake.

**Table 3:** Comparative analysis of younger masters athletes against older masters athletes.

Nutrient	Averages of All Studies 35–50 Years			Averages of All Studies > 50 Years			Percentage Difference (%) †		
	Combined	Male	Female	Combined	Male	Female	Combined	Male	Female
Energy (kJ/day)	9073	11,780	7485	8902	9919	7819	2	17	−4
Macronutrients									
Protein									
g/day	97	109	84	92	87	97	5	22	−14
g/kg	1.4	1.4	1.3	1.3	1.3	1.3	7	7	0
%E	16	15	16	17	16	20	-6	−6	−22
Fat									
g/day	87	104	73	77	82	71	12	24	3
g/kg	1.2	1.3	1.0	2.5	3.3	0.8	−70	−87	22
%E	34	36	33	30	33	28	13	9	16
Carbohydrate									
g/day	286	321	254	262	290	234	9	10	8
g/kg	3.9	3.7	3.8	3.9	3.9	4	0	−5	−5
%E	48	47	49	50	50	52	−4	−6	−6
Alcohol									
g/day	12	13	10	66	118	NR	−138	−160	-
%E	6	6	5	2	2	NR	100	100	-
Micronutrients (mg/day)									
Calcium	1230	1287	1097	1089	960	1153	12	29	−5
Magnesium	350	386	333	477	353	601	−31	9	−57
Iron	17	17	24	31	18	43	−58	−6	−57
Zinc	14	17	13	21	NR	21	−40	-	−47
Sodium	3179	3567	2921	2806	NR	2806	12	-	4

Macronutrient values have been presented mean ± standard deviation g/day; (g/kg BW/day); %E (percentage energy); † rounded to a whole number.

**Table 4:** Comparative analysis of masters athletes aged 35 to 50 years against the Australian population data.

Nutrient	Averages of All Studies 35–50 Years			Australian Health Survey 2011–2012			Percentage Difference (%) †		
	Combined	Male	Female	Combined	Male	Female	Combined	Male	Female
Energy (kJ/day)	9073	11,780	7485	8872	10,220	7540	2	14	−1
Macronutrients									
Protein									
g/day	97	109	84	94	108	80	3	1	5
g/kg	1.4	1.4	1.3	NR	NR	NR	-	-	-
%E	16	15	16	18	18	19	−12	−18	−17
Fat									
g/day	87	104	73	76	86	65	13	19	12
g/kg	1.2	1.3	1.0	NR	NR	NR	-	-	-
%E	34	36	33	31	31	31	9	15	6
Carbohydrate									
g/day	286	321	254	230	264	197	22	19	25
g/kg	3.9	3.7	3.8	NR	NR	NR	-	-	-
%E	48	47	49	43	43	44	11	9	11
Alcohol									
g/day	12	13	10	15	20	10	−22	−42	0
%E	6	6	5	4	5	4	40	18	22
Micronutrients (mg/day)									
Calcium	1230	1287	1097	834	911	758	38	34	37
Magnesium	350	386	333	351	393	309	0	−2	7
Iron	17	17	24	11	13	10	43	27	82
Zinc	14	17	13	11	13	9	24	27	36
Sodium	3179	3567	2921	2533	2915	2154	23	20	30

Macronutrient values have been presented mean ± standard deviation g/day; (g/kg BW/day); %E (percentage energy); † rounded to a whole number.

**Table 5:** Comparative analysis of masters athletes aged > 50 years against the Australian population data.

Nutrient	Averages of All Studies > 50 Years			Australian Health Survey 2011–2012			Percentage Difference (%) †		
	Combined	Male	Female	Combined	Male	Female	Combined	Male	Female
Energy (kJ/day)	8902	9919	7819	7792	8759	6919	13	12	12
Macronutrients									
Protein									
g/day	92	87	97	82	90	75	11	−3	26
g/kg	1.3	1.3	1.3	NR	NR	NR	-	-	-
%E	17	16	20	19	18	19	−11	−12	5
Fat									
g/day	77	82	71	66	73	59	15	12	18
g/kg	2.5	3.3	0.8	NR	NR	NR	-	-	-
%E	30	33	28	31	30	31	−3	10	−10
Carbohydrate									
g/day	262	290	234	201	227	178	26	24	27
g/kg	3.9	3.9	4.0	NR	NR	NR	-	-	-
%E	50	50	52	43	43	43	15	15	19
Alcohol									
g/day	66	118	NR	14	18	11	130	147	-
%E	2	2	NR	5	6	4	−86	−100	-
Micronutrients (mg/day)									
Calcium	1089	960	1153	729	754	707	40	24	48
Magnesium	477	353	601	315	340	292	41	4	69
Iron	31	18	43	11	12	10	95	40	125
Zinc	21	NR	21	10	11	9	71	-	80
Sodium	2806	NR	2806	2105	2363	1872	29	-	40

Macronutrient values have been presented mean ± standard deviation g/day; (g/kg BW/day); %E (percentage energy); † rounded to a whole number.

## Data Availability

No new data were created or analysed in this study. Data sharing is not applicable to this article.

## Supplementary Materials

The following supporting information can be downloaded at https://www.mdpi.com/article/10.3390/nu15234973/s1: Table S1: Sample search strategy conducted in MEDLINE.
