# Supplementary material for: Dietary Intake of Masters Athletes: A Systematic Review
Source: Nutrients. 2023 Nov 30;15(23):4973. doi: 10.3390/nu15234973 (PMC10708321; doi:10.3390/nu15234973)
Supplement: Supplementary file 1 [file nutrients-15-04973-s001.zip › nutrients-2725317-supplementary.pdf]

Table S1: Medline[‡] search strategy

#	Search Statement	Results
1	((Master* or former or #lder or senior or veteran* or middle-age* or elite or mature) adj1 athlet*).tw.	4361
2	((Master* or World) adj2 Games).tw.	142
3	1 or 2	4486
4	Sport/	31829
5	Endurance.tw.	32350
6	sport*.tw.	83472
7	Ultra-endurance.tw.	373
8	Sprint*.tw.	9687
9	((mixed or team or skill*) adj1 sport*).tw.	2717
10	run*.tw.	206666
11	((long or short or trail or marathon or orienteer*) adj2 run*).tw.	12408
12	jog*.tw.	2522
13	swim*.tw.	42936
14	row*.tw.	27813
15	soccer.tw.	9460
16	football.tw.	10004
17	(Track adj2 field).tw.	896
18	weight?lift*.tw.	989
19	power?lift*.tw.	237
20	wrestl*.tw.	2032
21	walk*.tw.	128252
22	"Body Build*".tw.	1206
23	skii*.tw.	2600
24	triathl*.tw.	1756
25	vigorous.tw.	27370
26	aerobic.tw.	90408
27	anaerobic.tw.	80035
28	((High or Low) adj1 intensity).tw.	43999

29	exercise.tw.	277260
30	"Physical* Activ*".tw.	129220
31	(Rugby adj1 (League or Union)).tw.	1661
32	((Habitual or chronic*) adj1 (exercise* or train*)).tw.	2084
33	Athletic*.tw.	18444
34	((strength or resistance) adj1 (train* or exercis*)).tw.	19349
35	Cycling.tw.	66844
36	Cyclist*.tw.	5603
37	4 or 5 or 6 or 7 or 8 or 9 or 10 or 11 or 12 or 13 or 14 or 15 or 16 or 17 or 18 or 19 or 20 or 21 or 22 or 23 or 24 or 25 or 26 or 27 or 28 or 29 or 30 or 31 or 32 or 33 or 34 or 35 or 36	1046387
38	3 and 37	3889
39	diet.tw.	346666
40	((Energy or diet* or nutri*) adj1 intake).tw.	55546
41	calori*.tw.	92353
42	Kilojoule*.tw.	311
43	M#cronutrient*.tw.	26046
44	Carbohydrate*.tw.	141555
45	Protein*.tw.	3204677
46	fat*.tw.	938234
47	vitamin*.tw.	221820
48	mineral*.tw.	185598
49	alcohol*.tw.	353954
50	(Diet* adj1 (quality or assessment or indicat* or pattern*)).tw.	19199
51	(("weigh* food" or Food or diet*) adj1 record*).tw.	6449
52	"Food diar*".tw.	1629
53	"Food frequency questionnaire*".tw.	13171
54	Questionnaire*.tw.	567493
55	Survey*.tw.	712884
56	"24 hour recall".tw.	1116
57	NRV.tw.	84
58	DRI.tw.	1378
59	AMDR.tw.	70

60	RDI.tw.	1606
61	RDA.tw.	3329
62	(Western adj1 (food* or diet*)).tw.	4281
63	Nutrit*.tw.	307557
64	Nutrient*.tw.	159433
65	fiber.tw.	164907
66	fibre.tw.	40407
67	39 or 40 or 41 or 42 or 43 or 44 or 45 or 46 or 47 or 48 or 49 or 50 or 51 or 52 or 53 or 54 or 55 or 56 or 57 or 58 or 59 or 60 or 61 or 62 or 63 or 64 or 65 or 66	6254568
68	38 and 67	1196

‡Via Ovid
